# By-degree Health and Economic Impacts of Lyme Disease, Eastern and Midwestern United States

**DOI:** 10.1007/s10393-024-01676-9

**Published:** 2024-03-13

**Authors:** Haisheng Yang, Caitlin A. Gould, Russ Jones, Alexis St. Juliana, Marcus Sarofim, Matt Rissing, Micah B. Hahn

**Affiliations:** 1 Abt Associates, 6130 Executive Boulevard, Rockville, MD 2085 USA; 2 Climate Change Division, Climate Science and Imapcts Branch, U.S. Environmental Protection Agency, 1200 Pennsylvania Ave NW, 4226-G South, Washington, DC 20460 USA; 3 Institute for Circumpolar Health Studies, University of Alaska-Anchorage, 3211 Providence Drive, Anchorage, AK 99508 USA

**Keywords:** Lyme disease, Vector-borne disease, Ticks, Climate change

## Abstract

Lyme disease (LD) is the most common vector-borne disease in the United States (U.S.). This paper assesses how climate change may influence LD incidence in the eastern and upper Midwestern U.S. and the associated economic burden. We estimated future *Ixodes scapularis* habitat suitability and LD incidence with a by-degree approach using variables from an ensemble of multiple climate models. We then applied estimates for present-day and projected habitat suitability for *I. scapularis,* present-day presence of *Borrelia burgdorferi*, and projected climatological variables to model reported LD incidence at the county level among adults, children, and the total population. Finally, we applied an estimate of healthcare expenses to project economic impacts. We show an overall increase in LD cases with regional variation. We estimate an increase in incidence in New England and the upper Midwestern U.S. and a concurrent decrease in incidence in Virginia and North Carolina. At 3°C of national warming from the 1986–2015 baseline climate, we project approximately 55,000 LD cases, a 38-percent increase from present-day estimates. At 6°C of warming, our most extreme scenario, we project approximately 92,000 LD cases in the region, an increase of 145 percent relative to current levels. Annual LD-related healthcare expenses at 3°C of warming are estimated to be $236 million (2021 dollars), approximately 38 percent greater than present-day. These results may inform decision-makers tasked with addressing climate risks, the public, and healthcare professionals preparing for treatment and prevention of LD.

**Supplementary Information:**

The online version contains supplementary material available at 10.1007/s10393-024-01676-9.

## Introduction

In the eastern United States (U.S.), *Ixodes scapularis* Say, or the eastern blacklegged tick, is the primary vector of *Borrelia burgdorferi* sensu stricto and *B. mayonii*, the pathogenic causative sources of Lyme disease (hereafter, “LD”) (Brownstein et al., 2005; Dolan et al., 2016). There are a multitude of symptoms associated with LD. Acute symptoms may include an erythema migrans rash, fever, chills, headache, fatigue, muscle and joint aches, and swollen lymph nodes. Days to months after a tick bite, and if symptoms are un- or under-treated, more severe sequelae can occur, including chronic neurodegenerative, rheumatological, and arthritic conditions (estimated in approximately 12% of U.S. cases; Halperin, 2013) and cardiac outcomes such as heart arrhythmia or Lyme carditis (estimated 1–10% of U.S. cases; Bush & Vazquez-Pertejo, 2018; Kullberg et al., 2020; Radesich et al., 2022). Health effects in children are comparable to those of adults, although the overall lifetime impact may be magnified given the potential for children to live for many more years than adults with life-altering conditions such as juvenile arthritis or carditis (Steere et al., 1977, 2016; Beach et al., 2020; Mac et al., 2020). Furthermore, illness may prevail as posttreatment Lyme disease syndrome (PTLDS) (CDC, 2022), which can manifest in a variety of ways including long-term cognitive effects, chronic fatigue, and muscular and joint pain (Steere et al., 2016; Wong et al., 2022).

Healthcare costs in the U.S. for LD vary and depend on the severity of illness (Adrion et al., 2015). Overall annual incidence in the U.S. approaches 106.6 cases per 100,000 individuals (Nelson et al., 2015). Nationwide studies determined that LD-related annual healthcare expenses, inclusive of short- and long-term medical care, have ranged from $345 million to $1.3 billion ($391 million to $1.88 billion in 2021; Adrion et al., 2015; Hook et al., 2022). Hospitalization rates are estimated by Bloch et al. (2022) as 6.98 per 100,000 cases annually. One study assessed median expenses as $11,688 (2016 dollars; approximately $13,234 in 2021 dollars) per patient, per hospitalization (Schwartz et al., 2020). Another study estimated mean LD hospitalization costs at $33,440 (2018–2019 dollars; approximately $35,229 in 2021 dollars) (Bloch et al., 2022). Non-hospitalization treatment estimates were far less (Zhang et al., 2006; Hook et al., 2022). Adrion et al. (2015) found the average cost of treatment as $2968 (2008 dollars; approximately $4282 in 2021 dollars), inclusive of inpatient and outpatient treatment. LD symptoms and healthcare expenses can have broad implications for individual patients. For instance, LD-related sequelae may lead to socioeconomic effects through healthcare costs or missed work (Johnson et al., 2011; Hirsch et al., 2018; Schwartz et al., 2020; Maxwell et al., 2022). There also may be a disproportionate burden on low-income, uninsured, or under-insured individuals who do not have sick leave or lack the means to seek care (Hirsch et al., 2018). Further, the aggregate healthcare costs for pediatric cases may be greater over their lifetime than adult cases, resulting from the potential for younger patients to live for many more years with chronic conditions.

There are fundamental connections between climate and LD incidence. Climate factors influence multiple drivers of LD incidence including the behaviors of the tick vector, reservoir hosts, and humans. Humid environments support adult tick survival by preventing desiccation (Eisen et al., 2016a, b; Ginsburg et al., 2017), although larval and nymphal *I. scapularis* can withstand colder, drier temperatures (Eisen et al., 2016a; Thomas et al., 2020). As climate change has increased temperatures and changes in precipitation patterns, there have been increases in the prevalence and geographic range of tick vectors, *B. burgdorferi*, and LD (Eisen et al., 2016a; Burtis et al., 2022; Zhang et al., 2022). Additionally, the prevalence and range of ticks and LD incidence are affected by climate-related changes in the behavioral traits of hosts that are suitable reservoirs for the spirochetes (e.g., rodents, raccoons, birds, humans, domesticated animals) (Ostfeld et al., 1995). For instance, climate change may alter the duration of time in which host species are active during conditions favorable to tick development and spirochete transfer, as well as the extent of the geographic range of hosts (Giardina et al., 2000; LoGiudice et al., 2003). Climate change also has been linked to a northward expansion in the habitat ranges of hosts that are not suitable reservoirs for *B. burgdorferi*, such as skinks (Giery & Ostfeld, 2007; Ginsburg et al., 2021). Changes in human behaviors (e.g., increased outdoor recreation earlier or later in the year, urban development patterns) over recent decades have led to an increase in humans encroaching on tick habitat, thus creating more opportunities for exposure to *I. scapularis* and *B. burgdorferi* (Diuk-Wasser et al., 2021; Hook et al., 2021; Kugeler et al., 2022).

Following approaches consistent with the U.S. Environmental Protection Agency’s (EPA 2021) Climate Change Impacts and Risk Analysis (CIRA; www.epa.gov/cira) 2.0 project (Martinich & Crimmins, 2019), this analysis advances the literature by projecting age-related incidence of LD along the East Coast and in the Northeast and Upper Midwest regions of the U.S. for multiple climate-warming scenarios and estimating healthcare costs following a by-degree approach. By describing potential impacts associated with a change in temperature, this analysis can inform public health interventions such as messaging to influence human behavior change, greenhouse gas mitigation decisions, education of healthcare practitioners and the public, implementation of vector surveillance and control activities, or vaccination campaigns, should an LD vaccine for children and adults become widely available (Beck et al., 2021; Eisen & Stafford, 2021; Tiffin et al., 2022).

## Methods

### Data Sources

#### Climate, Land Cover, and Elevation Data

We drew on multiple data sources to conduct this study. Baseline climate data were drawn from Livneh et al. (2015) for the years 1986–2005. For the baseline, we used a multi-year aggregation of precipitation and temperature data to create bioclimatic variables for estimating habitat suitability for *I. scapularis*, relying on the *dismo* and *modleR* packages in R [(R Version: “Spotted Wakerobin” Release (e7373ef8, 2022-09-06) for Windows; RStudio 2022.07.2 Build 576 (R Core Team, 2021)]. (The *dismo* package creates 19 bioclimatic variables to model temperature and precipitation variability. Find the full list of bioclimatic variables at https://github.com/cran/dismo/blob/master/R/biovars.R.) We used data from the 2016 National Land Cover Database to measure forest cover (U.S. Geological Survey [USGS], 2016). Elevation data also came from USGS (2018). This paper used a by-degree-of-warming approach: analyzing impacts by degree of climate change while holding constant socioeconomic variables, thus allowing for the construction of damage functions and clarified the influence of climate without confounding changes in other variables (Sarofim et al., 2021). For future climate, we used data to estimate six levels of warming, relying on a set of selected general circulation models (GCMs) using Representative Concentration Pathway (RCP) 8.5. The selection of these models and data were consistent with the CIRA framework and appear in Table 1.

**Table 1:** General Circulation Models and the Year Each Reaches Degrees of Warming for RCP 8.5: (Central Years for the 11-Year Time-Periods Used for Each GCM).

GCM (RCP 8.5)	1°C	2°C	3°C	4°C	5°C	6°C
CanESM2^*^	2011	2033	2048	2062	2076	2091
CCSM4^†^	2011	2037	2059	2077	2091	–
GISS E2 R^‡^	2025	2052	2082	–	–	–
HadGEM2 ES^§^	2013	2029	2044	2055	2064	2077
MIROC5^‖^	2017	2033	2050	2067	2081	
GFDL CM3^¶^	2013	2032	2049	2061	2071	2087

*Canadian Earth System Model second generation.

^†^Community Climate System Model version 4.

^‡^Goddard Institute for Space Studies version E2 R.

^§^Hadley Centre Global Environment Model version 2.

^‖^Model for Interdisciplinary Research on Climate version 5.

^¶^Geophysical Fluid Dynamics Laboratory Climate Model version 3.

This table demonstrates six General Circulation Models and different years for each degree of warming for RCP 8.5. Each cell represents the central year when the 11-year moving average of annual temperatures exceeds a given temperature threshold for that climate model.

#### Tick Distribution and Lyme Disease Incidence Data

The annual county-level incidence of LD from 2008 to 2019 was obtained through a data request to the U.S. Centers for Disease Control and Prevention’s (CDC) Division of Vector-Borne Diseases and the *National Notifiable Diseases Surveillance System*: *Lyme Surveillance Data 2008–2019* (CDC, 2022b). The dataset included LD incidence among children (defined in the dataset as ages 0–19) and adults (defined in the dataset as ages ≥ 20); however, in some cases, these data were missing or masked to protect personally identifiable health information where county-level counts were low and thus too small to ensure anonymity. We collapsed the case data at the county level by averaging across 2008–2019 to account for any years in which LD data were not available, and to account for any deviations that may have resulted from underreporting. For the baseline distribution of LD incidence by county, please reference Table A1 (Supplemental). In cases where LD cases were not broken into adult and child cases, we estimated this breakdown in two ways. When fewer than 25 percent of the data were missing, we distributed LD cases using the same distribution of reported cases for children and adults as in the baseline period. When more than 25 percent of the data were missing, we distributed the cases based on the national distribution of adult and child cases, 72 percent and 28 percent, respectively (Kugeler et al., 2022). County-level population counts for individuals aged 19 or younger, and individuals aged 20 or older, consistent with the age-ranges for the LD incidence data, were obtained from the *U.S. Census 5-Year American Community Survey* (U.S. Census, 2020). County-level information on the presence of *B. burgdorferi* and the presence of *I. scapularis* was sourced from the CDC (CDC, 2022b, 2022c). We considered *I. scapularis* “present” in a county when the tick was recorded as “established” in the CDC dataset (at least six ticks, or two or more life stages, were observed in a county within a 12-month period) (CDC, 2022b).

#### Healthcare Costs Data

We adjusted estimates from Adrion et al. (2015) to project the healthcare costs associated with LD. Adrion et al. (2015) considered medical claims for patients diagnosed with LD over a 12-month period and reported the average added healthcare costs compared to a control population. This estimate included medical insurance claims such as inpatient care, outpatient care, medications, laboratory testing, and other expenses.

#### Modeling Framework

**Figure 1:**
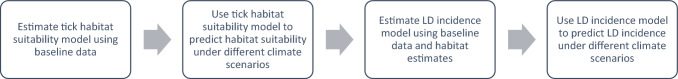
Process for Estimating Future LD Cases. *Notes*. This figure corresponds with the modeling framework outlined in the text. Estimating LD incidence proceeds in four steps. First, we estimated a present-day model for habitat suitability (Eq. 1). Second, we inputted future bioclimatic variables into the estimated baseline model to product future habitat suitability estimates. Third, we used the present-day habitat suitability estimates in our zero-inflated negative binomial model to estimate a model for the relationship between bioclimatic variables, habitat suitability, the presence of *B. burgdorferi*, and land-use variables (Eq. 2). Fourth, we inputted future bioclimatic variables into the estimated model to project the count of LD under future climate scenarios.

The analysis involved several steps for estimating future LD incidence and associated costs under different climate scenarios (Fig. 1).

First, we used the reported distribution of *I. scapularis* and present-day climate and land use (forest cover and elevation) data to develop a model for habitat suitability for the tick vector in counties in the Upper Midwest, Northeastern, and East Coast U.S. Next, we used estimates of current LD incidence at the county level to create a present-day LD incidence model based on the present-day habitat suitability estimated above, present climate, land use, human population, and the presence of *B. burgdorferi* in a county.The sample of states includes Connecticut, Delaware, District of Columbia, Illinois, Indiana, Iowa, Maine, Maryland, Massachusetts, Michigan, Minnesota, New Hampshire, New Jersey, New York, North Carolina, Ohio, Pennsylvania, Rhode Island, Vermont, Virginia, West Virginia, and Wisconsin.

To create future estimates of *I. scapularis* habitat suitability, we applied the coefficient estimates from a present-day habitat suitability model to the future bioclimatic variables produced by the six different climate models described above. To create future estimates of LD incidence, we applied the coefficients from the present-day LD model to the future bioclimatic variables, estimated future *I. scapularis* habitat suitability, and present-day land use, human population, and *B. burgdorferi* presence. We created a single LD estimate for a county by averaging across climate model results for each of the six temperature scenarios. Finally, we estimated associated economic costs by multiplying the number of predicted cases by the case-level cost for each additional case of LD. Each of these steps is described in more detail below.

#### Modeling Present-Day I. scapularis Habitat Suitability and LD Incidence

We used the following generalized linear model to project the habitat suitability of *I. scapularis,* for present and future climate scenarios:

1 $$ \Pr \left( {I.~scapulari\;s_{c} } \right) = ~\beta _{0} ~ + X^{\prime } \left( {{\text{Biovar}}\;s_{c} } \right) + \gamma ^{\prime } \left( {{\text{Land}}\;{\text{Use}}_{c} } \right) + \epsilon _{c}  $$

where the likelihood of *I. scapularis* in county *c* was modeled as a function of bioclimatic variables *Biovars* and terrestrial variables including forest cover and elevation (*Land Use*). To create a model of present-day habitat suitability, we used *modleR* to select the variables to estimate habitat suitability and validate our model at the county level. (A full list of bioclimatic variables appears in Table 2.)

**Table 2:** Bioclimatic and Land Use Variables Used in Habitat Suitability Model.

Variable name	Variable description
Bio1	Annual Mean Temperature
Bio2	**Mean Diurnal Range**
Bio3	Isothermality
Bio4	Temperature Seasonality
Bio5	Maximum Temperature of Warmest Month
Bio6	Minimum Temperature of Coldest Month
Bio7	Temperature Annual Range
Bio8	**Mean Temperature of Wettest Quarter**
Bio9	Mean Temperature of Driest Quarter
Bio10	**Mean Temperature of Warmest Quarter**
Bio11	Mean Temperature of Coldest Quarter
Bio12	Annual Precipitation
Bio13	Precipitation of Wettest Month
Bio14	Precipitation of Driest Month
Bio15	**Precipitation Seasonality**
Bio16	Precipitation of Wettest Quarter
Bio17	Precipitation of Driest Quarter
Bio18	**Precipitation of Warmest Quarter**
Bio19	Precipitation of Coldest Quarter
Forest Cover	**Percent of County with Forest Cover**
Elevation	**Elevation of County**

Selected variables for the present-day habitat suitability model are in bold.

This table presents the list of bioclimatic variables and land-use variables used in Eqs. (1, 2). Bolded variables were used in the present-day habitat suitability model. The variables used in future habitat suitability models appear in Table A5. For the list of bioclimatic variables, visit the World Clim Bioclimatic Variables webpage.

During the modeling process, we created five samples of the data, where each iteration estimated a model on four of the samples and validated against the leave-out sample. Each sample’s model produced a predicted probability of tick habitat suitability at the county level. The present-day habitat suitability results for each county and climate model in this analysis were the average of the predicted habitat suitability across the five samples.

We used our present-day habitat suitability estimates to estimate current LD incidence using the following zero-inflated negative binomial model. We used a zero-inflated model due to the number of counties *not* reporting any incidence of LD. We also tested our zero-inflated negative binomial model against a zero-inflated Poisson model. The log-likelihood test favors the zero-inflated negative binomial model over the zero-inflated Poisson model (Eq. 2), where we regressed the count of LD (i.e., LD incidence) for county *c* onto the predicted *I. scapularis* habitat suitability, the bioclimatic variables, and land-use variables described in Eq. (1). We included all the bioclimatic variables in Table 2 except for mean temperature of the coldest quarter and temperature annual range, which were omitted due to collinearity. We also included *B. burgdorferi* presence as an indicator for whether the spirochete has been detected in a county. We used this model to estimate the count of LD cases when LD has been detected in a county. We included human *Population* and *Habitat Suitability* as estimators in the model when LD had not been reported in a county. The variable *Lyme Disease* represented the previously detected presence of LD in a county.

2 $$ {\text{Lyme}}\;{\text{Disease}}_{c}  = \left\{ {\begin{array}{*{20}l}    {\beta _{0} ~ + \beta _{1} \left( {{\text{Habitat}}\;{\text{Suitability}}_{c} } \right) + ~\beta _{2} \left( {{\text{Population}}_{c} } \right) + \epsilon _{c} } \hfill & {{\text{if}}\;{\text{Lyme}}\;{\text{Disease}}_{c}  = 0} \hfill  \\    {\beta _{0} ~ + \beta _{1} \left( {B.~burgdorferi_{c} } \right) + ~X^{\prime } \left( {{\text{Biovar}}\;s_{c} } \right) + \gamma ^{\prime } \left( {{\text{Land}}\;{\text{Use}}_{c} } \right) + \epsilon _{c} } \hfill & {{\text{if}}\;{\text{Lyme}}\;{\text{Disease}}_{c}  > 0} \hfill  \\   \end{array} } \right.$$

### Modeling Future *Ixodes scapularis* Habitat Suitability Lyme Disease Incidence

To model future *I. scapularis* habitat suitability, we estimated a similar model as Eq. 1. Although we used the same parameters, we applied forward and backward variable selection to create the models to estimate future habitat suitability (for the selected variables, see Table A2, Supplementary). Like our methods to estimate present habitat suitability, we created five samples of the data, where each iteration estimated a model on four of the samples and validated against the leave-out sample. Each model then produced the predicted probability of future tick habitat suitability, where the final probability was the average across these five results.

The variable selection methods used to construct habitat suitability for present and future climate scenarios differ but produce similar results. For future habitat suitability, we relied on the *step* function in R, which uses the AIC to perform variable selection. For present habitat suitability, we relied on *modleR*, which selects variables based on correlation to other variables (i.e., removes highly correlated variables). The qualitative difference between these results was small; see Figure A1 (Supplemental) for a comparison of baseline results between the two methods.

To project LD incidence under future climate scenarios, we applied the parameter estimates from the present-day LD incidence model (Table A3, Supplemental) to the future bioclimatic variables, *B. burgdorferi* indicator, land-use variables, and projected likelihood of *I. scapularis* habitat suitability under future climate scenarios.

### Modeling Future Healthcare Costs of Lyme Disease

We adjusted the healthcare costs from Adrion et al. (2015) to find a case-level cost of $4282 (2021 dollars) using the medical care Consumer Price Index (U.S. Bureau of Labor Statistics 2021) from the midpoint of 2008. Since this estimate averaged costs of LD cases involving outpatient visits as well as those requiring inpatient care, it resulted in a lower per-patient estimate than those that focused only on the higher costs associated with hospitalization costs or long-term health outcomes such as PTLDS (e.g., certain components of Adrion et al., 2015; Schwartz et al., 2020). This lower per-patient cost was applied to more patients and thus was inclusive of a broader range of relatively short-term treatment options; such that all else being equal, the aggregate total costs were higher than for studies that focused only on cases requiring inpatient care or that included costs associated with PTLDS. However, these costs did not quantify other, potentially substantial costs associated with externalities such as lost workdays or productivity.

## Results

### *Ixodes scapularis* Habitat Suitability and Present-Day Lyme Disease Incidence

**Figure 2:**
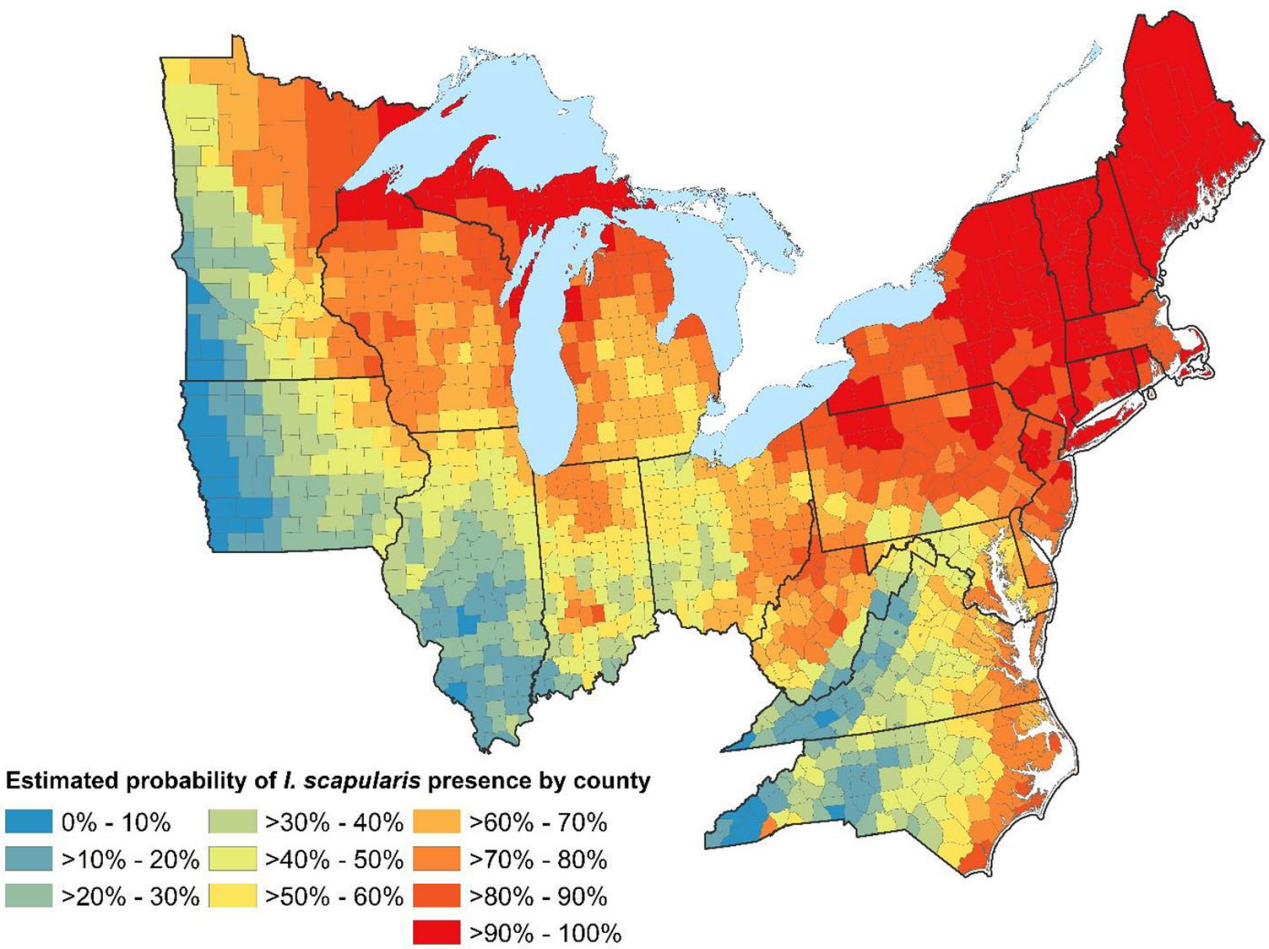
Present-Day Habitat Suitability for *I. scapularis* in the Northeast, East Coast, Upper Midwest United States. This figure shows the estimated present-day habitat suitability for *I. scapularis* by county in the Northeast, along the East Coast, and Upper Midwestern states. The model used to produce this graphic is shown in Table A4, in the supplementary materials. As is evidenced here, the estimated probability, reflected by darker shades of yellows, oranges, and reds, largely were concentrated in the northernmost locations.

Our present-day habitat suitability estimates for *I. scapularis* showed that areas in New England, the upper Midwest, and along the East Coast have the highest habitat suitability for *I. scapularis* (Fig. 2)*.* Notably, increases in the mean temperature of the warmest quarter and diurnal range led to a decrease in habitat suitability. However, increases in precipitation during the warmest quarter of the year and the percentage of the county with forest cover were associated with increased habitat suitability (Tables A2 and A4, Supplementary). The climate drivers of habitat suitability were similar in our future *I. scapularis* habitat suitability results. Given the negative association between temperature and habitat suitability, we observed a contraction of habitat suitability with each degree of warming (Table A5, Supplementary).

The geographic distribution of the predicted present-day *I. scapularis* habitat suitability is depicted in Figure 2. At baseline, the geographic distribution of LD was concentrated in the Northeast (predominantly northern New England), upper Midwest, and along the East Coast, with very little incidence in the lower Midwest (i.e., Iowa, Indiana, and Illinois).

**Figure 3:**
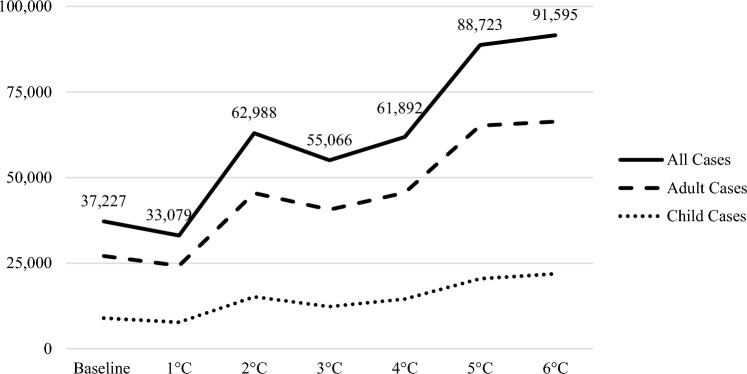
Predicted Annual LD Cases, by Temperature Bin and Lifestage (All Cases, Adult Cases, Child Cases). This graphic represents the predicted counts of adult LD cases, child LD cases, and total LD cases by degree-bin of warming. The counts for the total LD cases, adult LD cases, and child LD cases appear in Table 3.

During the baseline time-period of 1986–2015, we observed an estimated 27,000 annual adult cases and approximately 9000 child cases (Table 3 and Fig. 3).

**Table 3:** Estimated Number of LD Cases, Change in Case Numbers from the Baseline, Estimated Economic Costs, and Differences in Costs from Baseline at Each Degree of Temperature Change by Demographic Group.

Bin (degree-change)	Annual LD cases by degree			Change from baseline			Percent-change in LD cases			Estimated, projected annual economic costs	Difference in cost from baseline
	Adult (≥ 20 years)	Child (0–19 years)	Total	Adult (≥ 20 years)	Child (0–19 years)	Total	Adult (≥ 20 years)	Child (0–19 years)	Total		
Baseline	27,103	8921	37,227	–	–	–	–	–	–	$159,386,397	–
1°C	24,257	7737	33,079	− 2847	− 1184	− 4148	− 11%	− 11%	− 13%	$141,628,698	− $17,757,699
2°C	45,476	15,191	62,988	18,373	6270	25,761	69%	68%	70%	$269,684,272	$110,297,875
3°C	40,666	12,297	55,066	13,563	3377	17,840	48%	50%	38%	$235,767,628	$76,381,231
4°C	45,567	14,529	61,892	18,464	5608	24,666	66%	68%	63%	$264,992,474	$105,606,077
5°C	65,217	20,485	88,723	38,114	11,564	51,496	138%	141%	130%	$379,868,783	$220,482,386
6°C	66,355	21,889	91,595	39,251	12,968	54,369	145%	145%	146%	$392,166,946	$232,780,549

Each row shows the estimated LD cases, the change from the predicted baseline, percentage change from baseline, estimated economic costs, and differences in costs from baseline at each degree-change. Estimated LD cases, changes in case counts from baseline, and percent change are shown by the adult age group, the child age group, and total population. Annual LD cases by degree are visualized in Fig. 3.

### Future LD Incidence

**Figure 4:**
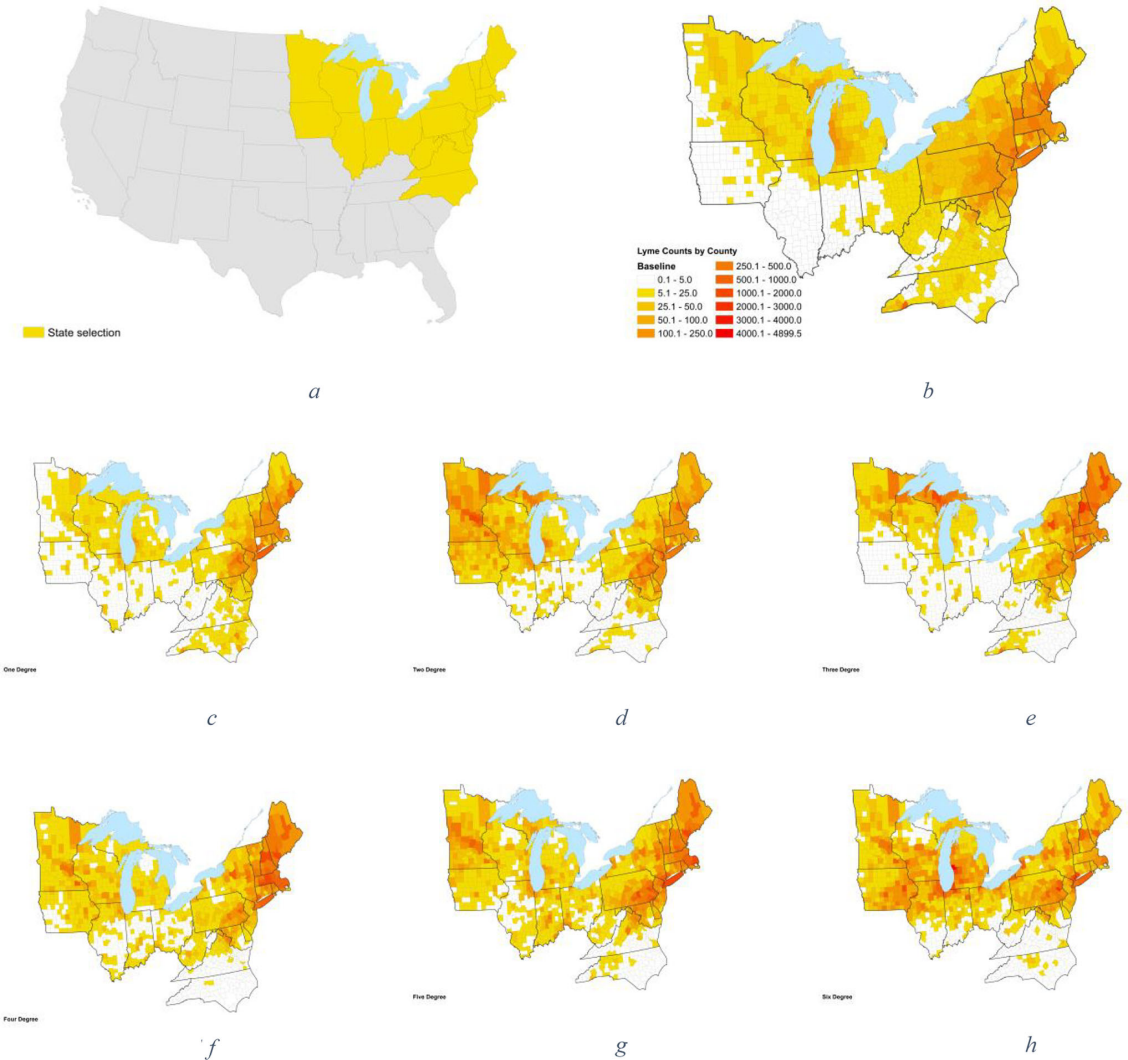
Geographic Distribution of Estimated County LD Cases, by Temperature Bins. The maps in this figure show the differences in estimated future geographic distribution of LD incidence, following the by-degree-of-warming approach employed in this analysis. Panel **a** depicts the states included in this analysis. Panel **b** represents present-day (baseline) temperatures and LD incidence. Panels **c** through **h** demonstrate changes in LD cases across our region of interest following different degree-increases in temperatures. (Panel **c** shows change in cases following 1°C of warming, Panel **d** shows change following 2°C of warming, and so forth.)

Our estimates of future LD incidence showed an increasing trend in the Eastern and upper Midwestern U.S. following more severe warming (Fig. 4), paralleling the present-day geographic distributions of habitat suitability and LD cases demonstrated in Figures 2 and 3, respectively. This was driven by increases in LD incidence in New England and upper Midwestern U.S. with concurrent decreases in LD incidence in Virginia and North Carolina. The trends for the incidence of LD among adults and children were similar, with the total number of cases among adults remaining higher than among children across all levels of warming. In terms of absolute numbers of cases, our model predicted approximately 55,000 cases of LD per year at 3°C, a decrease from the projected 63,000 annual cases at 2°C. At 3°C, we observed an estimated 41,000 annual LD cases in adults (48 percent increase relative to baseline) and approximately 12,000 annual cases in children (50 percent increase relative to baseline) (Table 3). However, in the 6°C warming climate scenario, we projected a 145 percent increase in cases from the baseline, which represents approximately 92,000 cases, or an increase of about 54,000 cases from baseline. For the model parameters used to project LD cases for different climate scenarios, please reference Table A6, in the supplementary materials.

### Present-Day and Future Healthcare Costs of Lyme Disease

During the baseline period, we estimated that current annual reported LD cases in the region of interest (approximately 37,000) represent approximately $145 million annually in healthcare costs, in 2021 dollars. Consistent with the other components of this analysis, annual collective, short-term inpatient and outpatient healthcare costs at 3°C of warming were estimated as $236 million in 2021 dollars, an increase of approximately $76 million greater than baseline expenses. At the highest temperature of 6°C, cost projections were approximately $392 million annually, in 2021 dollars (Table 3).

## Discussion

To the best of our knowledge, this is a first-of-its-kind analysis linking climate change with projected healthcare costs associated with LD along the East Coast, and in New England and Upper Midwest of the U.S., areas that presently experience the highest rates of this illness in the country (Kugeler et al., 2015). LD is likely to have a considerable impact on the health of thousands more children and adults over the coming decades across these regions, particularly in more northern areas, leading to tens to hundreds of millions of dollars in annual healthcare costs.

At the peak temperature explored in this study (6°C), we projected that climate change could increase inpatient and outpatient healthcare costs for LD by approximately $233 million annually relative to the present-day (2021 dollars). The subsequent costs to future generations of healthcare expenses, and lost work and wages, could have individual-level implications for social mobility, overall health, the healthcare industry, and the general economy (Hirsch et al., 2018).

The estimates of habitat suitability and LD incidence at baseline were consistent with prior work. Hahn et al. (2016) reported similar habitat suitability regions for *I. scapularis.* Schwartz et al. (2017) reported that between 2008 and 2015, 30,158 (reported in 2010) to 38,468 (reported in 2009) confirmed and probable cases LD cases were documented.

A notable caveat to these estimates is that we used reported LD cases for this analysis. These national estimates of costs likely are under-estimates, given that approximately only one in ten cases of LD is reported (Nelson et al., 2015; Cartter et al., 2018; Kugeler et al., 2022). Thus, our projections likely are under-estimates of future cases and healthcare costs. LD can have myriad health effects on individuals, irrespective of age, which can range from mild to severe, and thus can lead to a wide range of healthcare-related expenses. Thus, accurate reporting and accounting is critical for understanding the true burden of disease, including future healthcare costs, as we experience climate change.

Relying on the by-degree-of-warming approach, we show that from the baseline period, climate change and warming may increase LD incidence at temperatures up to 6°C. Consistent with previous studies (Brownstein et al., 2005; Kugeler et al., 2015; Ogden et al., 2018; Couper et al., 2021), our results show counties at the northern margins of the Northeast (predominantly New England) and Upper Midwest regions of the U.S. are projected to experience increases in LD cases, particularly among adults.

Interestingly, while within our region of interest, overall incidence is increasing, the range of vectors and pathogens, transmission patterns, and density and concentration of LD incidence rates are varying and, in some locales, declining. LD incidence in counties at the southern end of the current disease distribution (e.g., North Carolina, Virginia) is projected to experience an overall decline in cases. Ohio shows decreases in incidence for mild warming but increases at higher temperatures. That said, these results are not surprising given similar findings regarding LD spatial contraction within this region by Brownstein et al. (2005), Burtis et al. (2022), and Ginsburg et al. (2021). The projected declines likely are driven at least in part by a change in the range of suitable habitat for *I. scapularis* in the southeastern U.S. We hypothesize that such decreases in habitat suitability may be due to high temperatures driving and promoting tick diapause, or reductions in spirochete transmission due to changes in vector-host interactions and tick questing behaviors (Ogden et al., 2018; Elias et al., 2021).

Over the coming decades, climate change is likely to affect incidence rates of vector-borne diseases throughout the U.S. (McDermott-Levy et al., 2021; Baker et al., 2022). Already, warming temperatures, changes in precipitation patterns, and changes in habitat, range, and behaviors of vectors and hosts have led to the spread of diseases such as LD, Zika, and others throughout the U.S. (Beard et al., 2016). For this reason, it is important to consider the ramifications of the total costs of cases of LD now and in the future to provide a fuller picture of the risk to human health.

Potential effects on children’s health are worthwhile noting. While there are few differences in the manifestations of health effects in children versus adults, we stratify by age to demonstrate the numbers of children who may be affected in the future. In aggregate, children may experience greater effects and thus healthcare costs given the potential for longer life-years to live with adverse health outcomes. We anticipate that children with PTLDS will have greater lifetime healthcare costs as a result. Additional data on the incidence rates and costs of PTLDS and acute healthcare costs in children would allow more accurate projections.

This study has some limitations. The use of RCP8.5 does not imply a judgment regarding the likelihood of that scenario. The relationship between LD and temperature then could be interpreted in the context of any future scenario, as RCP8.5 encompasses the broadest range of possible future temperatures. Research has shown that 2°C of warming in RCP8.5 results in similar effects as those projected to result from 2°C of warming in other warming scenarios (Sarofim et al., 2021).

The underdiagnosis and underreporting of LD is a well-known issue (Nelson et al., 2015; Cartter et al., 2018; Kugeler et al., 2022). This may lead to an underestimation of current and future LD cases by our algorithm, as it has been trained on reported data. Additionally, there are some limitations to our habitat suitability and LD incidence models. Although our habitat modeling results are consistent to similar studies (Ogden et al., 2014, 2018; Burtis et al., 2022; Hahn et al., 2016), our predictions relied on the known presence of *I. scapularis* in a county. These data are obtained through vector surveillance efforts, which vary substantially by county. Our LD modeling framework does not account for factors such as tourism leading to LD diagnosis in a county different from the county where the infection took place (Chiu et al., 2011; Turrisi et al., 2021), the recent spread of ticks and their hosts into previously unsuitable habitat, or for increases in *B. burgdorferi* prevalence in the tick population. These factors may result in a new region with high LD risk.

Additionally, there are factors that this study has not considered that could lead to an increase in the total assessed impacts of climate on LD incidence. We choose to limit the geographic region for this analysis to states with historically high incidence of LD. However, it is likely that LD risk will expand beyond these regions under future climate change. In addition, we did not estimate economic impacts from LD cases in the western U.S. The study does not consider different subpopulations of *I. scapularis*, or evolution of future subspecies, that may have questing behaviors or other characteristics that lead to more ability to adapt to climate changes (Arsnoe et al., 2015, 2019; Ginsburg et al., 2014, 2017). We rely on present-day estimates of forest cover for the present-day and future habitat suitability and LD models. As such, this study does not capture the potential expansion of suburban areas well-suited for host species in the future, which may increase human-tick encounters (Keesing et al., 2009). Human behavior and susceptibility may be affected by future education measures, tick control efforts, public health measures, or vaccine development, leading to a reduction in LD incidence (e.g., Eisen, 2021; Behler et al., 2020; Poland, 2001). Finally, there are additional health effects of *B. burgdorferi* (such as PTLDS) and other tick-borne diseases (e.g., anaplasmosis, babesiosis, rickettsia) that are not addressed in the present study and could increase healthcare costs. Our dataset does not reflect diagnosis nor treatment received via primary care providers or hospitalization; thus, we apply a single cost estimate of direct medical costs to encompass all related expenses.

## Conclusion

This study estimates the change in LD incidence in the Northeast, Upper Midwest, and along the East Coast U.S. due to climate change, including age-related effects and estimates of healthcare costs. The presentation of the results using a by-degree framework adds to the body of work that EPA has developed, and thus, this research can be used in applications that require damage functions, and makes the results broadly applicable. We project that climate warming likely will lead to a notable regional increase in LD incidence and associated healthcare costs, especially when aggregated across the Midwest and Northeastern U.S., and predominantly in northern New England. This is despite projected overall decreases in LD incidence rates in more southerly areas that may experience higher temperatures. These results may serve to inform policymakers tasked with addressing climate risks, the U.S. public, and healthcare professionals who are preparing for treatment and prevention of LD.

### Supplementary Information

Below is the link to the electronic supplementary material.

Supplementary file1 (PDF 9 KB)

Supplementary file2 (PDF 116 KB)

Supplementary file3 (PDF 60 KB)

Supplementary file4 (PDF 22 KB)

Supplementary file5 (PDF 63 KB)

Supplementary file6 (PDF 17 KB)

Supplementary file7 (PDF 61 KB)

Supplementary file8 (PDF 22 KB)
