# Supplementary material for: By-degree Health and Economic Impacts of Lyme Disease, Eastern and Midwestern United States
Source: Ecohealth. 2024 Mar 13;21(1):56–70. doi: 10.1007/s10393-024-01676-9 (PMC11127817; doi:10.1007/s10393-024-01676-9)
Supplement: Supplementary file 1 — Supplementary file1 (PDF 9 KB) [file 10393_2024_1676_MOESM1_ESM.pdf]

Supplemental Material

By-degree health & economic impacts of Lyme disease, Eastern & Midwestern United States

Haisheng Yang, Caitlin A. Gould, Russ Jones, Alexis St. Juliana, Marcus Sarofim, Matt Rissing, Micah Hahn

Table of Contents

Figure A1. Comparing Baseline Habitat Suitability, Using Different Variable Selection Methods

Table A1. Baseline Incidence Rate of LD, by State

Table A2. Model Coefficients to Construct Future Habitat Suitability

Table A3. Model Estimates for Baseline LD Incidence

Table A4. Model Coefficients to Construct Present Habitat Suitability

Table A5. Bioclimatic Variables and Predicted Tick Probability, by Temperature Bin

Table A6. Model Coefficients to Construct Future Habitat Suitability
