# Supplementary material for: By-degree Health and Economic Impacts of Lyme Disease, Eastern and Midwestern United States
Source: Ecohealth. 2024 Mar 13;21(1):56–70. doi: 10.1007/s10393-024-01676-9 (PMC11127817; doi:10.1007/s10393-024-01676-9)
Supplement: Supplementary file 2 — Supplementary file2 (PDF 116 KB) [file 10393_2024_1676_MOESM2_ESM.pdf]

Supplementary – Figure A1. Comparing Baseline Habitat Suitability, Using Different Variable Selection Methods

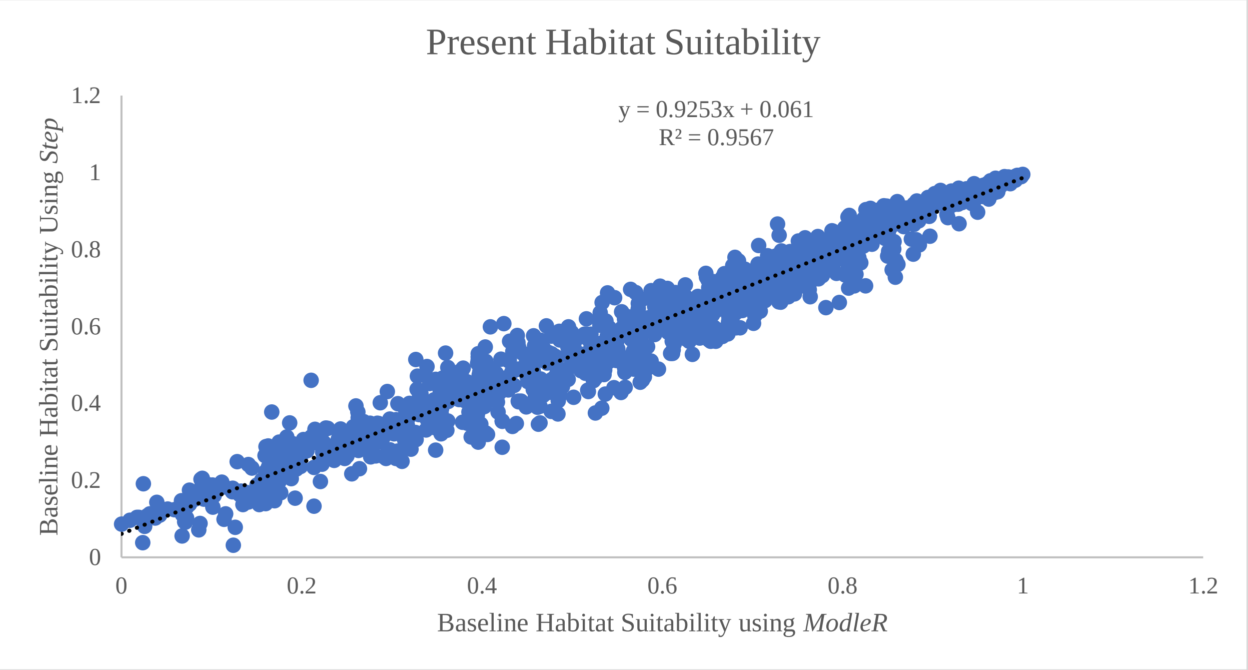

Caption. This figure compares baseline habitat suitability estimates produced by *modleR* and the *step* function in R. As evidenced, there is very strong linearity shown between the two functions.
