# Supplementary material for: By-degree Health and Economic Impacts of Lyme Disease, Eastern and Midwestern United States
Source: Ecohealth. 2024 Mar 13;21(1):56–70. doi: 10.1007/s10393-024-01676-9 (PMC11127817; doi:10.1007/s10393-024-01676-9)
Supplement: Supplementary file 3 — Supplementary file3 (PDF 60 KB) [file 10393_2024_1676_MOESM3_ESM.pdf]

Supplementary – Table A1. Baseline Incidence Rate of LD, by State

	Number of Counties	Mean Annual Cases	Standard Deviation	Min	Max
Connecticut	8	234.89	91.06	143.09	355.36
Delaware	3	188.12	150.53	94.00	361.73
District of Columbia	1	66.00	N/A	66.00	66.00
Illinois	102	2.75	7.84	0.00	61.45
Indiana	92	1.00	2.90	0.00	16.45
Iowa	99	2.42	5.64	0.00	38.45
Maine	16	83.91	80.55	6.50	287.45
Maryland	24	68.37	60.62	6.88	226.27
Massachusetts	14	230.9	172.88	43.13	586.22
Michigan	83	3.07	5.65	0.00	27.60
Minnesota	87	21.05	37.45	0.00	219.45
New Hampshire	10	125.61	138.67	8.25	396.91
New Jersey	21	190.76	121.78	28.09	489.55
New York	62	72.80	81.44	0.00	327.73
North Carolina	100	2.91	4.73	0.00	23.80
Ohio	88	3.56	5.60	0.00	24.00
Pennsylvania	67	116.77	107.99	11.71	528.45
Rhode Island	5	136.91	90.26	49.44	270.91
Vermont	14	49.79	47.71	0.00	125.45
Virginia	133	9.74	24.57	0.00	197.27
West Virginia	55	12.69	23.71	0.00	168.00
Wisconsin	72	33.05	24.31	5.00	128.91

Caption. This table presents the average incidence of LD during the baseline period across states included in the sample. Additionally, we provide information on the number of counties in a state, the minimum number of cases in a state, the maximum number of cases in a state, and the number of counties in a state or the District of Columbia.
