# Supplementary material for: By-degree Health and Economic Impacts of Lyme Disease, Eastern and Midwestern United States
Source: Ecohealth. 2024 Mar 13;21(1):56–70. doi: 10.1007/s10393-024-01676-9 (PMC11127817; doi:10.1007/s10393-024-01676-9)
Supplement: Supplementary file 5 — Supplementary file5 (PDF 63 KB) [file 10393_2024_1676_MOESM5_ESM.pdf]

Supplementary – Table A3. Model Estimates for Baseline LD Incidence

Variable Name	Description	(1)	(2)
		Count Model	Zero Count Model
Bio1	Annual Mean Temperature	-6.68***<br>(0.78)	
Bio2	Mean Diurnal Range	-1.61**<br>(0.81)	
Bio3	Isothermality	0.50*<br>(0.27)	
Bio4	Temperature Seasonality	-0.07***<br>(0.01)	
Bio5	Maximum Temperature of Warmest Month	0.63<br>(0.50)	
Bio6	Minimum Temperature of Coldest Month	-0.41<br>(0.25)	
Bio8	Mean Temperature of Wettest Quarter	0.00<br>(0.02)	
Bio9	Mean Temperature of Driest Quarter	-0.01<br>(0.02)	
Bio10	Mean Temperature of Warmest Quarter	6.35***<br>(0.96)	
Bio12	Annual Precipitation	0.46***<br>(0.07)	
Bio13	Precipitation of Wettest Month	-2.22***<br>(0.34)	
Bio14	Precipitation of Driest Month	-1.65***<br>(0.47)	
Bio15	Precipitation Seasonality	0.34***<br>(0.04)	
Bio16	Precipitation of Wettest Quarter	-0.42**<br>(0.19)	
Bio17	Precipitation of Driest Quarter	1.27***<br>(0.28)	
Bio18	Precipitation of Warmest Quarter	-0.32***<br>(0.12)	
Bio19	Precipitation of Coldest Quarter	-0.24<br>(0.23)	
Elevation	Elevation of County	-0.00***<br>(0.00)	
Forest Cover	Percent of County with Forest Cover	0.64**<br>(0.32)	
B. burgdorferi	Indicator for Presence of *B. burgdorferi*	0.63***<br>(0.09)	
Pr(*Scapularis*)	Habitat Suitability for *I. scapularis*		-3.59***<br>(0.34)
Population	County Population		-0.00***<br>(0.00)

Intercept	Model Constant	-24.83***	2.72***
		(8.11)	(0.23)
**N**	Observations	1,156	1,156

Caption. This table shows the coefficient estimates associated with Equation 2. Standard errors reported in parenthesis. Coefficients statistically significant at *0.10, **0.05, and ***0.01.
