# Supplementary material for: By-degree Health and Economic Impacts of Lyme Disease, Eastern and Midwestern United States
Source: Ecohealth. 2024 Mar 13;21(1):56–70. doi: 10.1007/s10393-024-01676-9 (PMC11127817; doi:10.1007/s10393-024-01676-9)
Supplement: Supplementary file 6 — Supplementary file6 (PDF 17 KB) [file 10393_2024_1676_MOESM6_ESM.pdf]

Supplementary - Table A4. Model Coefficients to Construct Present Habitat Suitability

**Variable Name**	**Description**	**Model 1**	**Model 2**	**Model 3**	**Model 4**	**Model 5**
Intercept	Model Constant	17.72*** (1.62)	18.32*** (1.71)	16.67*** (1.63)	17.52*** (1.64)	16.76*** (1.58)
Bio2	Mean Diurnal Range	-0.44*** (0.12)	-0.48*** (0.12)	-0.51*** (0.12)	-0.39*** (0.12)	-0.42*** (0.12)
Bio8	Mean Temperature of Wettest Quarter	0.10*** (0.03)	0.08** (0.04)	-0.02* (0.01)	0.08** (0.03)	0.08** (0.03)
Bio10	Mean Temperature of Warmest Quarter	-0.77*** (0.08)	-0.77*** (0.08)	-0.68*** (0.08)	-0.76*** (0.08)	-0.70*** (0.08)
Bio15	Precipitation Seasonality	-0.04*** (0.01)	-0.03** (0.01)	0.06* (0.03)	-0.04*** (0.01)	-0.03*** (0.01)
Bio18	Precipitation of Warmest Quarter	0.45*** (0.08)	0.46*** (0.08)	0.46*** (0.08)	0.45*** (0.08)	0.40*** (0.07)
Forest Cover	Percent of County with Forest Cover	1.85*** (0.55)	2.31*** (0.55)	2.52*** (0.56)	2.01*** (0.54)	1.88*** (0.54)
Elevation	Elevation of County	-0.00*** (0.00)	-0.00*** (0.00)	-0.00*** (0.00)	-0.00*** (0.00)	-0.00*** (0.00)

Notes. This table shows the coefficient estimates associated with Equation 1 for the models used to construct baseline habitat suitability estimates. Standard errors reported in parenthesis. Coefficients statistically significant at *0.10, **0.05, and ***0.01.
