# Supplementary material for: By-degree Health and Economic Impacts of Lyme Disease, Eastern and Midwestern United States
Source: Ecohealth. 2024 Mar 13;21(1):56–70. doi: 10.1007/s10393-024-01676-9 (PMC11127817; doi:10.1007/s10393-024-01676-9)
Supplement: Supplementary file 7 — Supplementary file7 (PDF 61 KB) [file 10393_2024_1676_MOESM7_ESM.pdf]

Supplementray – Table A5. Bioclimatic Variables and Predicted Tick Probability, by Temperature Bin

	Temperature Bin						
	Baseline	1°C	2°C	3°C	4°C	5°C	6°C
Pr(*I. scapularis*)	0.57	0.33	0.21	0.10	0.07	0.04	0.01
Annual Mean Temperature	10.23	11.45	12.46	13.55	14.55	15.58	16.67
Mean Diurnal Range	11.72	11.98	12.02	12.16	12.32	12.42	12.27
Isothermality	32.00	32.14	31.70	31.76	32.26	32.39	31.06
Temperature Seasonality	950.70	968.07	978.23	991.91	994.35	996.35	1051.40
Maximum Temperature of Warmest Month	28.79	30.61	31.92	33.28	34.39	35.67	37.42
Minimum Temperature of Coldest Month	-8.14	-6.96	-6.25	-5.28	-4.07	-2.89	-2.27
Temperature Annual Range	36.93	37.57	38.17	38.56	38.46	38.56	39.69
Mean Temperature of Wettest Quarter	19.38	18.08	18.85	17.53	19.16	20.24	20.52
Mean Temperature of Driest Quarter	0.83	3.38	5.12	8.44	9.92	11.31	15.85
Mean Temperature of Warmest Quarter	21.73	22.96	24.15	25.32	26.42	27.54	29.24
Mean Temperature of Coldest Quarter	-1.77	-0.72	0.17	1.05	2.09	3.28	3.75
Annual Precipitation	33.30	34.08	34.76	35.16	35.94	35.70	36.20
Precipitation of Wettest Month	3.87	4.20	4.30	4.31	4.49	4.47	4.75
Precipitation of Driest Month	1.77	1.67	1.68	1.71	1.76	1.68	1.63
Precipitation Seasonality	19.41	21.60	21.56	21.23	22.00	21.74	24.11
Precipitation of Wettest Quarter	10.75	11.07	11.23	11.31	11.58	11.54	12.07
Precipitation of Driest Quarter	5.93	6.04	6.13	6.26	6.33	6.32	6.25
Precipitation of Warmest Quarter	10.13	10.11	9.96	9.63	10.09	9.57	9.25

Precipitation of Coldest Quarter	6.19	6.59	6.96	7.46	7.68	7.56	8.25

Notes. This table shows the average habitat suitability and bioclimatic variable values at each temperature bin, starting at baseline.
