# Supplementary material for: By-degree Health and Economic Impacts of Lyme Disease, Eastern and Midwestern United States
Source: Ecohealth. 2024 Mar 13;21(1):56–70. doi: 10.1007/s10393-024-01676-9 (PMC11127817; doi:10.1007/s10393-024-01676-9)
Supplement: Supplementary file 8 — Supplementary file8 (PDF 22 KB) [file 10393_2024_1676_MOESM8_ESM.pdf]

Supplementary - Table A6. Model Coefficients to Construct Future Habitat Suitability

Variable Name	Description	Model 1	Model 2	Model 3	Model 4	Model 5
Intercept	Model Constant	21.54*** (2.28)	20.97*** (2.46)	17.49*** (2.56)	17.60*** (2.15)	17.77*** (2.32)
Bio3	Isothermality			-0.12** (0.06)		
Bio5	Maximum Temperature of Warmest Month	-0.95*** (0.09)	-0.90*** (0.1)	-0.62*** (0.11)	-0.72*** (0.07)	-0.79*** (0.1)
Bio8	Mean Temperature of Wettest Quarter	0.14*** (0.04)	0.08* (0.05)			0.08** (0.04)
Bio9	Mean Temperature of Driest Quarter			0.05 (0.03)		
Bio12	Annual Precipitation	0.17*** (0.04)	0.29*** (0.09)	0.23** (0.11)	0.30*** (0.09)	0.35*** (0.09)
Bio13	Precipitation of Wettest Month		0.73 (0.52)		0.81 (0.53)	
Bio16	Precipitation of Wettest Quarter		-0.89*** (0.32)	-0.77*** (0.22)	-1.31*** (0.30)	-0.61*** (0.20)
Bio17	Precipitation of Driest Quarter	0.51** (0.25)		0.72* (0.37)		
Bio18	Precipitation of Warmest Quarter		0.48** (0.21)	0.77*** (0.18)	0.82*** (0.18)	0.30 (0.19)
Bio19	Precipitation of Coldest Quarter	-0.64*** (0.24)	-0.36** (0.17)	-0.97** (0.38)	-0.38** (0.17)	-0.49*** (0.17)
Forest Cover	Percent of County with Forest Cover	1.60*** (0.44)	1.72*** (0.45)	2.28*** (0.57)	1.59*** (0.45)	1.43*** (0.44)
Elevation	Elevation of County	-0.002*** (0.0002)	-0.0022*** (0.0002)	-0.0018*** (0.0003)	-0.0022*** (0.0002)	-0.002*** (0.0002)

Notes. This table shows the coefficient estimates associated with Equation 1 for the models used to construct future habitat suitability estimates. Standard errors reported in parenthesis. Coefficients statistically significant at *0.10, **0.05, and ***0.01.
